# Cannabinoid Receptor 1 Regulates Zebrafish Renal Multiciliated Cell Development via cAMP Signaling

**DOI:** 10.3390/jdb13020020

**Published:** 2025-06-17

**Authors:** Thanh Khoa Nguyen, Sophia Baker, Julienne Angtuaco, Liana Arceri, Samuel Kaczor, Bram Fitzsimonds, Matthew R. Hawkins, Rebecca A. Wingert

**Affiliations:** Department of Biological Sciences, Center for Stem Cells and Regenerative Medicine, Center for Zebrafish Research, University of Notre Dame, Notre Dame, IN 46556, USA; sbaker9@nd.edu (S.B.); skaczor@nd.edu (S.K.); bfitzsim@nd.edu (B.F.); mhawkin7@nd.edu (M.R.H.)

**Keywords:** multiciliated cell, endocannabinoid pathway, cannabinoid receptor, Cnr1, cAMP signaling, kidney, ciliogenesis

## Abstract

Endocannabinoid signaling plays a significant role in neurogenesis and nervous system physiology, but its roles in the development of other tissues are just beginning to be appreciated. Previous reports have shown the presence of the key endocannabinoid receptor Cannabinoid receptor 1 (CB1 or Cnr1) in multiciliated (MCC) tissues and its upregulation in kidney diseases, yet the relationship between Cnr1 and renal MCC development is unknown. Here, we report that Cnr1 is essential for cilia development across tissues and regulates renal MCCs via cyclic AMP (cAMP) signaling during zebrafish embryogenesis. Using a combination of genetic and pharmacological studies, we found that the loss of function, agonism and antagonism of *cnr1* all lead to reduced mature renal MCC populations. *cnr1* deficiency also led to reduced cilia development across tissues, including the pronephros, ear, Kupffer’s vesicle (KV), and nasal placode. Interestingly, treatment with the cAMP activator Forskolin (FSK) restored renal MCC defects in agonist-treated embryos, suggesting that *cnr1* mediates cAMP signaling in renal MCC development. Meanwhile, treatment with the cAMP inhibitor SQ-22536 alone or with *cnr1* deficiency led to reduced MCC populations, suggesting that *cnr1* also mediates renal MCC development independently of cAMP signaling. Our findings indicate that *cnr1* has a critical role in controlling renal MCC development both via cAMP signaling and an independent pathway, further revealing implications for ciliopathies and renal diseases.

## 1. Introduction

The endocannabinoid signaling pathway is critical for the healthy development and function of many physiological systems. The pathway includes the two main receptors, CB1 and CB2, which are both G protein-coupled receptors (GPCR), sharing about 48% amino acid sequence identity, as well as their numerous endogenous ligands, called “endocannabinoids” [1,2]. Despite the similarities in sequence, the CB1 and CB2 receptors are expressed in widely different tissues. The CB1 receptor, also known as Cnr1 or CB1R, is highly expressed in the nervous system, including the hippocampus, cerebellum, basal ganglia, neocortex and brain stem [3]. Additionally, the CB1 receptor is expressed in the ovary, kidney, gastrointestinal (GI) tract, liver, muscle, adipose tissue and also vasculature [4,5,6,7,8,9,10,11,12]. On the other hand, the CB2 receptor is expressed predominantly in tissues involved in immune regulation including the thymus, spleen, GI tract and bone marrow [7,8].

Emerging research has suggested a significant role of the CB1 receptor in kidney development and disease. The CB1 receptor is expressed in adult kidney populations that include the glomeruli, afferent and efferent arterioles, proximal tubule, distal tubule and collecting duct in mammals [13,14,15,16]. The presence of the CB1 receptor has also been found across experimental models, including human kidney, human cell culture, murine and zebrafish [13,14,15,17,18,19,20]. To date, the CB1 receptor has also been implicated in a number of kidney diseases. For example, the expression level of the CB1 receptor was highly upregulated in the glomeruli in diabetic nephropathy, a leading cause of chronic kidney diseases worldwide [1]. CB1 expression was also highly upregulated in other kidney conditions, including renal fibrosis, acute interstitial nephritis and IgA nephropathy [17]. However, we currently still have limited knowledge about the developmental genetics and signaling pathways through which the CB1 receptor governs kidney formation, especially how the CB1 receptor regulates renal cell composition. In addition, the majority of the studies on CB1 in kidney diseases to date have been performed using murine models.

Meanwhile, other experimental paradigms, such as zebrafish, offer many advantages to study kidney ontogeny and disease. The many benefits of using zebrafish to study renal biology include high fecundity, embryonic transparency, high genetic conservation with humans, robust regeneration and rapid external development [21]. The zebrafish embryonic kidney, or pronephros, is also structurally conserved compared to the human kidney, yet contains only two nephrons compared to about almost a million nephrons in humans [22,23]. Specifically, each zebrafish nephron consists of a blood filter that is followed by a segmented tubule that modifies the filtrate with unique functional regions, including the proximal convoluted tubule (PCT), proximal straight tubule (PST) and distal early (DE) and distal late (DL) segments, which have corresponding physiological roles in mammals [22]. Additionally, a healthy zebrafish embryonic kidney has MCCs, which, in humans, have only been found in fetal and kidney-diseased conditions [24,25,26,27,28]. Interestingly, the CB1 receptor is expressed in MCCs in several tissues, including the MCCs of the cat oviduct or the rat brain ependymocytes [29,30]. Previous studies have reported expression of the CB1 receptor in tissues rich in MCCs in zebrafish, such as the brain and kidney [1,18]. However, we still do not know the role of Cnr1 in the MCCs of the kidney, as well as other ciliated tissues such as ear or nasal placode.

Here, we identified an essential role of CB1, also known as Cnr1 in zebrafish, in renal MCC development via genetic deficiency and pharmacological approaches. Both *cnr1* genetic loss of function, as well as agonism or antagonism of *cnr1* using pharmacological treatments, resulted in pericardial edema between 48 and 72 h post fertilization (hpf) along with delayed PCT coiling, indicating dysfunctional renal clearance due to MCC defects. Indeed, *cnr1* deficiency resulted in a decreased number of both mature and progenitor renal MCCs populations. Meanwhile, treatment with either *cnr1* agonists or antagonists resulted in a decreased number of only mature renal MCCs. We further discovered disrupted cilia development in the pronephros, KV, nasal placode and ear due to *cnr1* deficiency. Interestingly, renal MCC deficiency in agonist-treated embryos was rescued with the co-treatment of FSK, an adenylyl cyclase activator that promotes cAMP signaling. Additionally, treatment with SQ-22536, an adenylyl cyclase inhibitor, both alone and in tandem with *cnr1* deficiency, led to a reduction in renal MCC populations. Taken together, our study highlights that *cnr1* is essential for cilia development across zebrafish tissues and elucidates that *cnr1* regulates renal MCC development both independently and through cAMP signaling pathways.

## 2. Materials and Methods

### 2.1. Ethics Statement and Zebrafish Husbandry

The zebrafish in our studies were maintained by the Center for Zebrafish Research at the University of Notre Dame. Our experiments were approved under protocol numbers 19-06-5412 and 22-07-7335 by the University of Notre Dame Institutional Animal Care and Use Committee (IACUC).

### 2.2. Animal Models

Our work used the Tübingen strain zebrafish for the experiments. We raised and staged zebrafish following previously described methods [31]. For all experiments, we incubated embryos in E3 medium at 28 °C until the desired stage, treated them with 0.02% tricaine and then fixed them using either Dent’s solution (80% methanol, 20% DMSO) or 4% paraformaldehyde/1× PBS (PFA) [32]. We performed WISH experiments in biological triplicate, each of which has a sample size greater than 10 embryos. A minimum of 3 samples were quantified per experimental group for cilia data analysis.

### 2.3. Whole Mount in Situ Hybridization (WISH)

We performed WISH following previously published methods [32,33,34]. Linearized plasmids were transcribed in vitro with T7, T3 or SP6 enzymes to create antisense RNA probes either digoxigenin-labeled (*odf3b*, *cetn4*, *pax2a*, *jag2b*, *myl7*, *slc20a1a*, *trpm7*, *slc12a1*, *slc12a3*) or fluorescein-labeled (*odf3b*) via in vitro transcription using IMAGE clone templates as previously described [35,36,37].

### 2.4. Morpholino Knockdown

Morpholino oligonucleotides (MOs) were obtained from Gene Tools, LLC, Philomath, USA and suspended in DNase/RNase free water to create 4 mM stock solutions, which were stored at −20 °C. Embryos were injected at the one-cell stage with 3 nanoliters (nl) of diluted MO. The *cnr1* MO sequence is 5′-CTAGAGGAAACCTGTCGGAGGAAAT-3′, which has been previously validated in zebrafish [38,39].

### 2.5. Immunofluorescence (IF)

Whole-mount IF experiments were performed as previously described [40,41,42,43]. For cilia, anti-tubulin acetylated diluted 1:400 (Sigma) was used. For basal bodies, anti-γ-tubulin diluted 1:400 (Sigma, St. Louis, MO, USA) was used. For apical membrane, anti-aPKC diluted 1:500 (Santa Cruz Biotechnology, Santa Cruz, CA, USA) was used (Table S1).

### 2.6. Drug Treatments

Drug treatments were performed as previously described [44]. Chemicals were dissolved in DMSO to make a 5 mM or 10 mM stock solution, aliquoted and stored at −80 °C. Then, a 1 mM working solution dissolved in DMSO was made from a stock solution. The specific treatment solutions of each chemical used in this study were diluted to their desired concentration in E3. Drug treatments were performed on 6-well plates for embryos beginning at the shield stage (6 hpf) until the desired timepoints for study. For embryos studied over multi-day periods, drug solutions were refreshed at the beginning of each day. For WISH analysis, at the desired timepoints, the drug solution was removed, and embryos were washed two times with E3, then euthanized with 0.02% tricaine and fixed in 4% PFA for study. The dosage of each chemical used was chosen based on maximum penetration and minimal morphological defects. To ensure that the effects of agonists and antagonists on MCC development were only due to their effects, WT control embryos for each experiment were incubated with a similar volume of treatment solution including only DMSO. Treatments were performed in biological triplicate, with a minimum of 10 embryos per replicate.

### 2.7. PCT Phenotype Scoring

To score PCT coiling defects, we picked representative images of WT samples stained with the PCT marker *slc20a1a* at each timepoint of interest. We scored this level of coiling at each timepoint as “normal”. If PCT coiling level was not as developed compared to “normal”, samples would be scored as “delayed”.

### 2.8. Image Acquisition

We took live images and images of WISH samples using a Nikon Eclipse Ni with a DS-Fi2 camera. We measured WISH segment length with the Nikon Elements software polyline tool. IF images were acquired using a Leica Stellaris 8 DIVE confocal microscope. For WISH pictures, images were taken at 4× and 10×. For IF images, images were taken at 63×.

### 2.9. Quantification and Statistical Analysis

We used ImageJ/Fiji for all of our cilia measurements at a 63× magnification. The multi-point tool was used for counting. The segment line tool was used to measure cilia lengths in both proximal and distal segments. A minimum of 6 cilia length measurements were performed per sample for a minimum of 3 samples per experimental group. The plot profile function was used to measure fluorescent intensity. The average and standard deviation were calculated from measurements and unpaired t-tests, or one-way ANOVA were performed to compare measurements between experimental groups using GraphPad Prism 10 software. For binary classification data, we utilized Fisher’s exact test with Holm’s method for correcting for multiplicity (when necessary) [45]. All binary classification analysis was performed in R (v4.4.0) and significance for the adjusted *p*-value was set to *p* < 0.05.

## 3. Results

### 3.1. Loss of cnr1 Leads to Phenotypes Consistent with Renal MCC Defects

**Figure 1:**
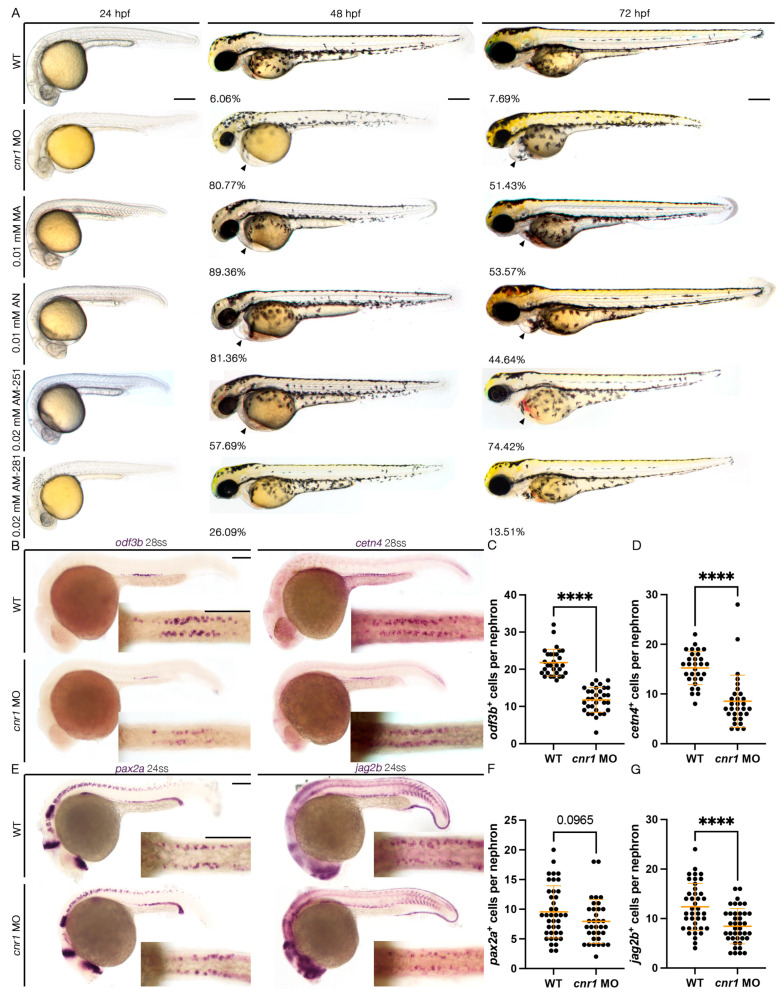
**Loss of *cnr1* leads to reduction in both mature and progenitor MCC populations:** (**A**) live imaging between different treatment groups between 24 and 72 hpf with the percentage of embryos with edema between 48–72 hpf (arrows indicate pericardial edema); scale bar = 200 μm; (**B**) 28 ss WT, *cnr1* MO stained via WISH using the mature MCC marker *odf3b* and *cetn4*; scale bar = 50 μm; (**C**,**D**) number of *odf3b^+^* and *cetn4^+^* cells per nephron at 28 ss; (**E**) 24 ss WT, *cnr1* MO stained via WISH using the progenitor MCC marker *pax2a* and *jag2b*; scale bar = 50 μm; (**F**,**G**) number of *pax2a^+^* and *jag2b^+^* cells per nephron at 24 ss. Data presented on graphs are represented as mean ± SD; **** *p* < 0.0001 (*t*-test).

To study the role of Cnr1 in zebrafish renal development, we utilized a combination of both genetic and pharmacological methods to target the Cnr1 receptor. First, to generate a genetically deficient model of Cnr1, we utilized a MO that effectively blocks the translation of the Cnr1 protein by blocking the translation of the *cnr1* mRNA, the effect of which has been previously reported in disrupting neuronal development in zebrafish [38,39]. To assess the effect of the *cnr1* MO on body morphology, we microinjected the *cnr1* MO at the single-cell stage and performed live brightfield imaging between 24 and 72 hpf. We observed that *cnr1* morphants developed progressive pericardial edema between 48 and 72 hpf, which often indicates dysfunctional renal clearance due to cilia defects (Figure 1A). Furthermore, since renal MCCs assist with fluid flow, which plays an important role in PCT morphogenesis and cell migration [22], we assessed PCT coiling using the segment marker *slc20a1a* between 48 and 72 hpf. Interestingly, we observed an increase in the number of embryos with delayed PCT coiling in *cnr1* morphants compared to WT embryos at both timepoints (Figure S1A–C).

To study the effect of the *cnr1* MO in renal MCC development, we performed WISH using markers *odf3b* and *cetn4* to assess mature MCC populations at the 28-somite stage (ss). We found that *cnr1* morphants exhibited a decrease in the number of both *odf3b^+^* and *cetn4^+^* cells per nephron compared to WT embryos (Figure 1B–D). We also assessed MCC progenitor populations using the two MCC progenitor markers, *pax2a* and *jag2b*, at 24 ss. We observed a significant decrease in the number of *jag2b^+^* cells per nephron and a slight decrease in the number of *pax2a^+^* cells per nephron in *cnr1* morphants compared to WT embryos (Figure 1E–G). Our results thus demonstrate that *cnr1* plays a significant role in zebrafish renal MCC development and function.

### 3.2. Cnr1 Agonism Leads to Phenotypes Consistent with Renal MCC Defects

To further explore the effects of *cnr1* function, we next employed a pharmacological approach to both activate and block Cnr1. To activate Cnr1, we used two drugs, Anandamide (AN) and Methanandamide (MA). AN, also known as AEA or ANA, is the first identified endocannabinoid, while MA is a synthetic analog of AN that has a higher affinity for Cnr1 [5,46]. Using concentrations of 0.01 mM MA and 0.01 mM AN, we performed drug treatments beginning at the shield stage, or 6 hpf, until the desired timepoints for analysis. We observed that treatment with either 0.01 mM MA or 0.01 mM AN led to development of pericardial edema from 48 to 72 hpf, indicative of dysfunctional clearance due to cilia defects (Figure 1A). Interestingly, when we assessed PCT coiling using the PCT marker *slc20a1a* between 48 and 72 hpf, we observed almost normal coiling with both 0.01 mM MA and 0.01 mM AN treatment at 48 hpf but significantly delayed at 72 hpf compared to WT embryos (Figure S1A–C).

**Figure 2:**
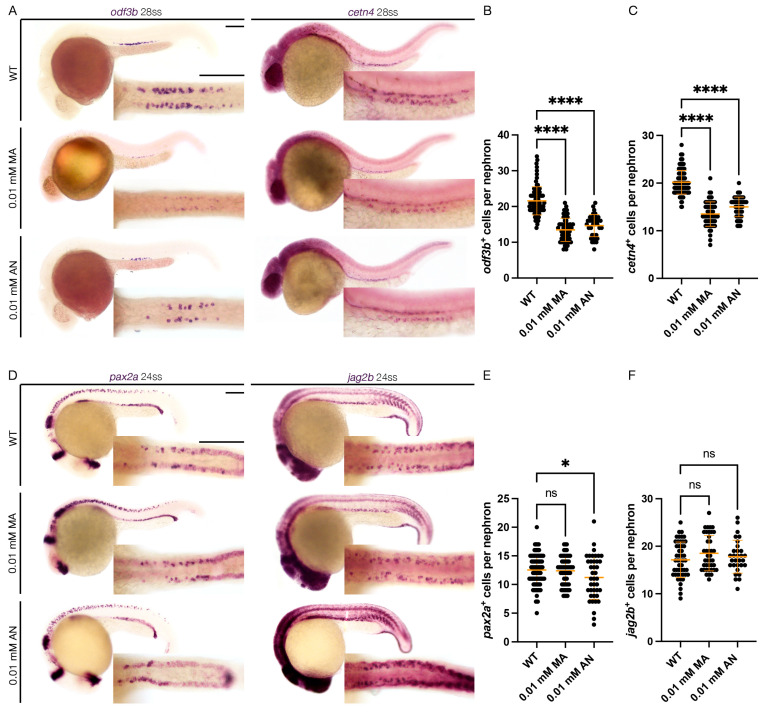
***cnr1* agonism leads to reduction in mature MCC population:** (**A**) 28 ss WT, 0.01 mM MA and 0.01 mM AN-treated embryos stained via WISH using the mature MCC marker *odf3b* and *cetn4*; scale bar = 50 μm; (**B**,**C**) number of *odf3b^+^* and *cetn4^+^* cells per nephron at 28 ss; (**D**) 24 ss WT, 0.01 mM MA and 0.01 mM AN-treated embryos stained via WISH using the progenitor MCC marker *pax2a* and *jag2b*; scale bar = 50 μm; (**E**,**F**) number of *pax2a^+^* and *jag2b^+^* cells per nephron at 24 ss. Data presented on graphs are represented as mean ± SD; * *p* < 0.05 and **** *p* < 0.0001 (ANOVA).

To assess the effect of Cnr1 agonism on renal MCC development, we performed WISH using mature MCC markers, *odf3b* and *cetn4* at 28 ss. We observed that both treatment with 0.01 mM MA and 0.01 mM AN led to a decreased number of both *odf3b^+^* and *cetn4^+^* cells per nephron compared to WT embryos (Figure 2A–C). We also assessed MCC progenitor populations using the two MCC progenitor markers, *pax2a* and *jag2b*, at 24 ss. We only observed a slight decrease in the number of *pax2a^+^* cells per nephron with the 0.01 mM AN treatment, but no significant difference between the number of *pax2a^+^* cells per nephron between 0.01 mM MA and WTs. Similarly, we observed non-significant differences in the number of *jag2b^+^* cells per nephron between the 0.01 mM MA and 0.01 mM AN treatments and WTs (Figure 2D–F). These results indicate that Cnr1 agonism leads to a reduction in mature MCCs but not the normal development of progenitor MCC populations.

### 3.3. Cnr1 Antagonism Leads to Phenotypes Consistent with Renal MCC Defects

Next, to block activity of Cnr1, we examined the effects of two drugs, AM-251 and AM-281, both of which are widely used Cnr1 antagonists [47]. Using the concentrations of 0.02 mM AM-251 and 0.02 mM AM-281, we performed drug treatment at the shield stage until the desired timepoints for analysis. Interestingly, we observed that treatment with 0.02 mM AM-251, but not 0.02 mM AM-281, led to the development of pericardial edema from 48 to 72 hpf, indicative of dysfunctional clearance due to cilia defects (Figure 1A). When we assessed PCT coiling using the PCT marker *slc20a1a* between 48 and 72 hpf, we observed almost normal coiling at 48 hpf and significantly delayed coiling at 72 hpf compared to WTs with 0.02 mM AM-251 treatment, but normal coiling at both stages with 0.02 mM AM-281 treatment (Figure S1A–C).

**Figure 3:**
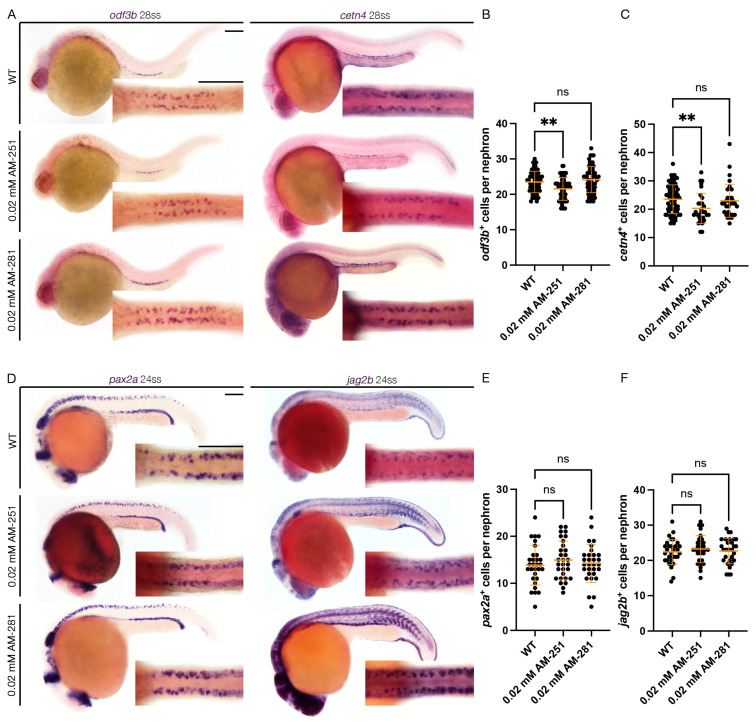
***cnr1* antagonism leads to reduction in mature MCC population:** (**A**) 28 ss WT, 0.02 mM AM-251 and 0.02 mM AM-281-treated embryos stained via WISH using the mature MCC marker *odf3b* and *cetn4*; scale bar = 50 μm; (**B**,**C**) number of *odf3b^+^* and *cetn4^+^* cells per nephron at 28 ss; (**D**) 24 ss WT, 0.02 mM AM-251 and 0.02 mM AM-281-treated embryos stained via WISH using the progenitor MCC marker *pax2a* and *jag2b*; scale bar = 50 μm; (**E**,**F**) number of *pax2a^+^* and *jag2b^+^* cells per nephron at 24 ss. Data presented on graphs are represented as mean ± SD;** *p* < 0.01 (ANOVA).

Next, to assess the effect of Cnr1 antagonism in renal MCC development, we performed WISH using mature MCC markers, *odf3b* and *cetn4,* at 28 ss. We observed that treatment with 0.02 mM AM-251 resulted in a decrease in the number of both *odf3b^+^* and *cetn4^+^* cells per nephron compared to WT embryos (Figure 3A–C). On the other hand, treatment with 0.02 mM AM-281 led to no significant differences between the number of *odf3b^+^* and *cetn4^+^* cells per nephron compared to WT embryos (Figure 3A–C). We also assessed MCC progenitor populations using the two MCC progenitor markers, *pax2a* and *jag2b*, at 24 ss. We did not observe any significant differences between the number of both *pax2a^+^* and *jag2b^+^* cells per nephron between WT, 0.02 mM AM-251 and 0.02 mM AM-281 treatment (Figure 3D–F). We concluded that treatment with *cnr1* antagonism, in this case only AM-251, leads to a reduction in the number of mature MCCs, but not the number of progenitor MCCs.

### 3.4. Assessing Renal Segmental Differences with cnr1 Deficiency, Cnr1 Agonism and Antagonism

As mentioned, zebrafish kidney segments include the PCT, PST and DE and DL segments [22]. MCCs are distributed in a salt and pepper pattern throughout the proximal and distal tubule and most highly concentrated in the PCT, PST and DE segments [22,48]. Each nephron segment expresses a distinctive set of genes [35]. As we observed a reduction in the number of mature MCCs due to *cnr1* deficiency, Cnr1 agonism and Cnr1 antagonism, we next investigated what happens to each of the pronephric segments. We thus performed WISH at 28 ss to survey each of the four segments, PCT labeled by *slc20a1a*, PST labeled by *trpm7*, DE labeled by *slc12a1* and DL labeled by *slc12a3*, between WT and each experimental condition. With the *cnr1* MO, we observed an increase in the domain length of *slc20a1a,* a slight increase in the domain length of *slc12a1* and a decrease in the domain length of *trpm7* and *slc12a3* compared to WTs (Figure S1D–H). Our results suggest that in addition to the regulation of renal MCC populations, *cnr1* may also play a role in regulating both proximal and distal renal cell fates.

With Cnr1 agonism, treatment with 0.01 mM MA led to a reduction in the DE segment labeled by *slc12a1* compared to WTs, but no significant difference in the domain length of *slc20a1a*, *trpm7* or *slc12a3* compared to WTs (Figure S2A–E). On the other hand, treatment with 0.01 mM AN led to a reduction in the PST segment labeled by *trpm7* compared to WTs, but no significant difference in the domain length of *slc20a1a*, *slc12a1* and *slc12a3* (Figure S2A,F–I). We conclude that other than the effect of MA on the DE segment, Cnr1 agonism has little effect on kidney segmentation.

With Cnr1 antagonism, treatment with 0.02 mM AM-251 led to a slight increase in the PCT segment labeled by *slc20a1a* compared to WTs, but no significant difference in the domain length of *trpm7*, *slc12a1* or *slc12a3* compared to WTs (Figure S3A–E). Treatment with 0.02 mM AM-281 did not result in any significant difference in any of the kidney segments compared to WTs (Figure S3A,F–I). We conclude that Cnr1 antagonism has little effect on kidney segmentation.

### 3.5. cnr1 Deficiency Leads to Reduced Cilia Development Across Zebrafish Tissues

**Figure 4:**
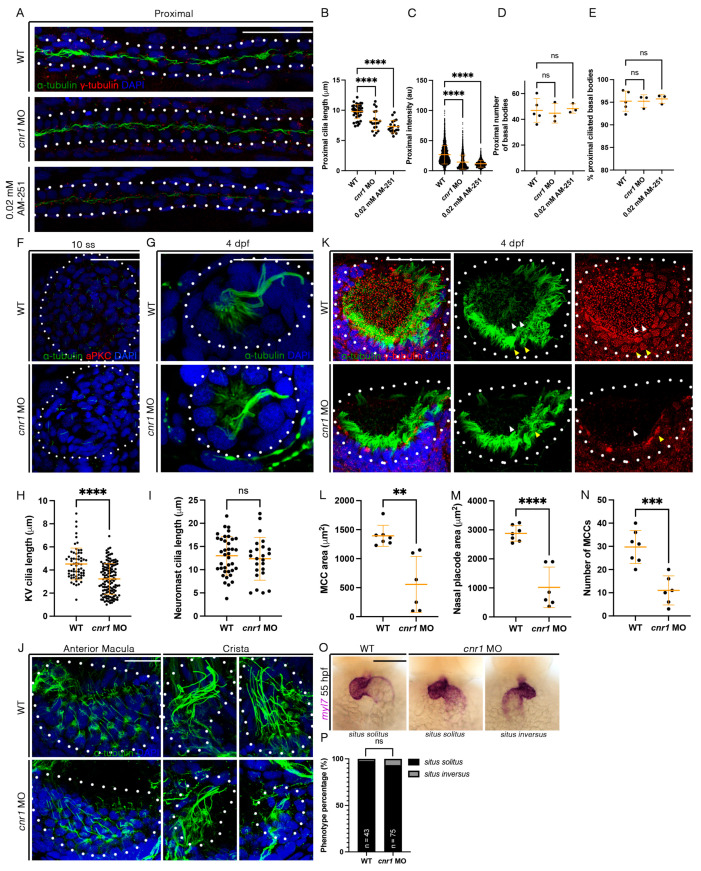
**Loss of *cnr1* leads to cilia defects across embryonic tissues:** (**A**) 28 hpf whole-mount IF for acetylated α-tubulin (cilia, green), γ-tubulin (basal bodies, red) and DAPI (nucleus, blue) in the proximal segment of WT, *cnr1* MO and embryos treated with 0.02 mM AM-251; scale bar = 50 μm; (**B**) proximal cilia length at 28 hpf; (**C**) fluorescence intensity plot of α-tubulin intensity within the proximal segment at 28 hpf; (**D**) number of basal bodies in the proximal segment at 28 hpf; (**E**) percentage of ciliated basal bodies/total basal bodies in the proximal segment at 28 hpf; (**F**) 10 ss whole-mount IF for acetylated α-tubulin (cilia, green), anti-PKC (membrane boundary, red) and DAPI (nucleus, blue) in the KV of WT and *cnr1* MO embryos; scale bar = 50 μm; (**G**) 4 dpf whole-mount IF for acetylated α-tubulin (cilia, green) and DAPI (nucleus, blue) in the neuromast of WT and *cnr1* MO embryos; scale bar = 25 μm; (**H**) KV cilia length at 10 ss; (**I**) neuromast cilia length at 4 dpf; (**J**) 4 dpf whole-mount IF for acetylated α-tubulin (cilia, green) and DAPI (nucleus, blue) in the anterior macula and crista of WT and *cnr1* MO embryos; scale bar = 25 μm; (**K**) 4 dpf whole-mount IF for acetylated α-tubulin (cilia, green), γ-tubulin (basal bodies, red) and DAPI (nucleus, blue) in the nasal placode of WT and *cnr1* MO embryos; scale bar = 25 μm; (**L**,**M**) nasal MCC area and nasal placode area at 4 dpf; (**N**) number of nasal MCCs at 4 dpf; (**O**) 55 hpf WT and *cnr1* MO embryos stained via WISH using heart marker *myl7;* scale bar = 50 μm; (**P**) phenotype percentage of heart looping phenotypes at 55 hpf. Data presented on graphs are represented as mean ± SD; ** *p* < 0.01 *** *p* < 0.001 and **** *p* < 0.0001 (*t*-test (**H**,**I**,**L**–**N**), ANOVA (**B**–**E**) and Fisher’s exact test (**P**)).

Previous studies have indicated that *cnr1* is expressed in ciliated tissues, most notably in the MCCs of the cat oviduct as well the brain ependymal cells in rats [29,30]. Given our observed phenotype with a reduction in mature MCCs in embryos treated with 0.02 mM AM-251 and both mature and progenitor MCC states in the pronephros of *cnr1* morphants, we next investigated whether the reduction in cilia development occurs across ciliated tissues. First, we performed whole-mount IF to detect α-tubulin, which labels cilia, and γ-tubulin to label basal bodies in the pronephros. Compared to WTs, *cnr1* morphants and embryos treated with 0.02 mM AM-251 exhibited a significant reduction in cilia length, as well as intensity of the α-tubulin signal in the proximal pronephros occupied by MCCs (Figure 4A–C) and the distal pronephros, which is mostly occupied with monociliated cells (Figure S4A–C). Interestingly, there were no significant differences in the number of basal bodies and the percentage of ciliated basal bodies in both the proximal and distal segment (Figure 4A,D,E and Figure S4A,D,E). Previous studies have also indicated no significant differences in cilia length and percentage of ciliated basal bodies between WTs and control MOs [41].

Next, we used whole-mount IF to examine cilia development in the KV, an important embryonic organ that serves important roles in L/R patterning. We observed a significant decrease in KV cilia length in *cnr1* morphants compared to WTs at 10 ss (Figure 4F,H). We also investigated cilia in the neuromast, a sensory hair cell organ of the lateral line system. Interestingly, there were no significant differences in cilia length between WT and *cnr1* morphants at 4 dpf (Figure 4G,I). Additionally, we surveyed cilia and basal bodies in the nasal placode. The zebrafish nasal placode cilia development includes a lateral rim rich in MCCs and an inner core rich in monociliated cells [21]. MCCs have been detected in the nasal placode as early as 18 hpf and frequently from 48 hpf and beyond [49,50,51,52,53].

As we observed a significant decrease in renal MCCs, we hypothesized that the nasal MCCs are also affected. Indeed, we observed a reduction in the area of nasal MCC coverage and the nasal placode area, as well as a reduction in the number of nasal MCCs in *cnr1* morphants compared to WTs at 4 dpf (Figure 4K–N). Furthermore, we observed defects in the ear cilia development in *cnr1* morphants, including stunted cilia growth in the anterior macula and crista compared to WTs at 4 dpf (Figure 4J). Finally, given our observation that KV cilia are reduced in length in *cnr1* morphants, we questioned whether the reduction in KV cilia could be associated with overt morphological defects due to L/R patterning, such as cardiac looping. To investigate this, we performed WISH with WT and *cnr1* morphants at 55 hpf using *myl7*, a cardiac marker. We observed a higher but non-significant percentage of *cnr1* morphants with randomized heart looping displaying the *situs inversus* phenotype than WTs (Figure 4O,P). Taken together, our studies indicate that *cnr1* is essential for ciliated cell development across zebrafish embryonic tissues.

### 3.6. Forskolin Rescues Renal MCC Reduction in Cnr1 Agonist-Treated Embryos

**Figure 5:**
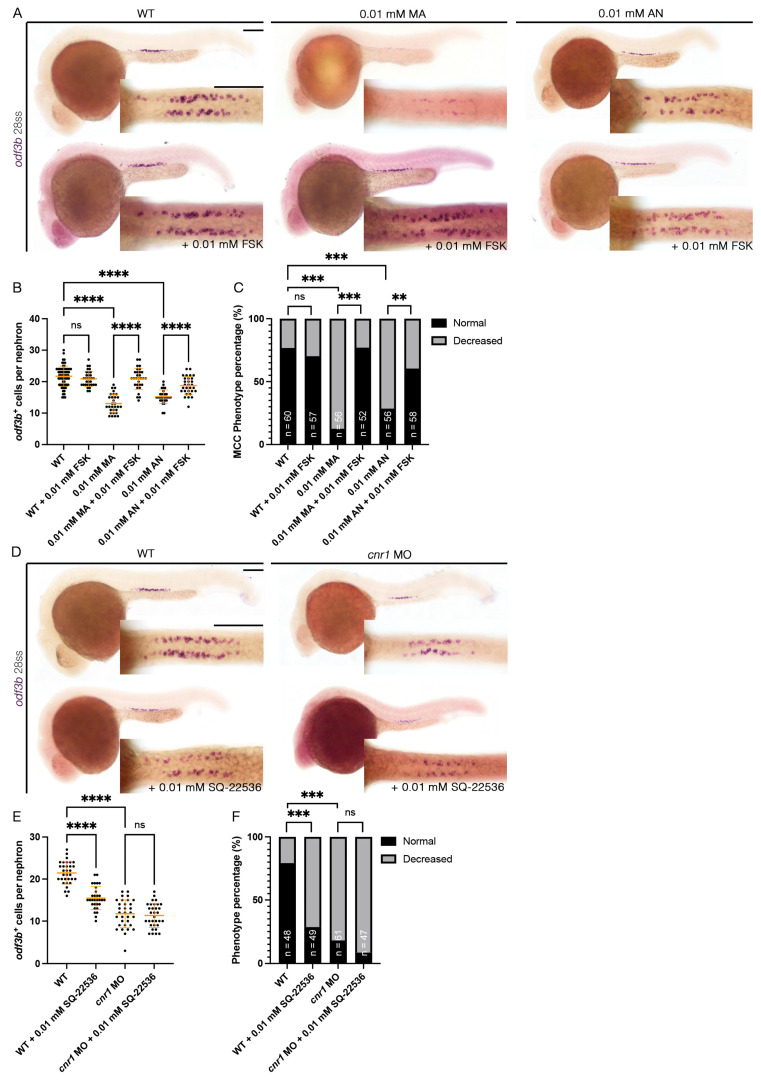
***cnr1* regulates renal MCC development via cAMP signaling and independently**: (**A**,**D**) 28 ss embryos between experimental groups stained via WISH using the mature MCC marker *odf3b*; scale bar = 50 μm; (**B**,**E**) number of *odf3b^+^* cells per nephron at 28 ss; (**C**,**F**) phenotype percentage of MCC phenotypes at 28 ss. Data presented on graphs are represented as mean ± SD; ** *p* < 0.01 *** *p* < 0.001 and **** *p* < 0.0001 (ANOVA (Figure 4B,E) and Fisher’s exact test with Holm’s correction (Figure 4C,F)).

Cnr1 has been known as a GPCR that inhibits the activity of adenylyl cyclase and thus, eventually, the production of cAMP [1]. Interestingly, previous studies have shown that cAMP is essential for ciliogenesis. For example, *ep4* is one of the GPCRs that increases the level of cAMP, and *ep4* morphants exhibited ciliogenesis defects such as hydrocephalus, laterality defects and reduction in KV cilia length. Interestingly, the addition of FSK, an adenylyl cyclase activator, partially rescued KV cilia defects and laterality defects [54]. Given our observation that overactivation of *cnr1* with agonists led to a decrease in renal MCC development, we hypothesized that *cnr1* activation led to the reduction in cAMP signaling that governs renal MCC development. To investigate this possibility, we performed a rescue experiment by co-treatment of Cnr1 agonists and FSK. To generate agonist-treated embryos, we performed drug treatment with either 0.01 mM MA or 0.01 mM AN at the shield stage until 28 ss. To generate rescued embryos, we performed drug treatment with either a combination of 0.01 mM MA and 0.01 mM FSK or a combination of 0.01 mM AN and 0.01 mM FSK at the shield stage until 28 ss. Using the MCC marker *odf3b*, we observed, as expected, a decrease in the number of *odf3b^+^* cells per nephron with the 0.01 mM MA and 0.01 mM AN treatment group, as well as an increase in ratio of embryos with a decreased MCC phenotype compared to WTs (Figure 5A–C). Interestingly, co-treatment with either a combination of 0.01 mM MA and 0.01 mM FSK or 0.01 mM AN and 0.01 mM FSK rescued the number of *odf3b^+^* cells per nephron, as well as the ratio of embryos with a decreased MCC phenotype, back to WT levels (Figure 5A–C). Treatment of 0.01 mM FSK alone neither led to an increase in the number of *odf3b^+^* cells per nephron nor an increase in embryos with the normal MCC phenotype compared to WTs (Figure 5A–C). Our results indicate that *cnr1* likely governs renal MCC development through cAMP signaling.

### 3.7. cnr1 Likely Governs Renal MCC Development via Additional Pathways

In addition to our observation that *cnr1* agonism leads to a reduction in renal MCCs in the pronephros, we also observed a reduction in renal MCCs in our *cnr1* morphants, which are deficient in Cnr1 protein (Figure 1B–G). As we found that *cnr1* governs renal MCC development through cAMP signaling, *cnr1* might also influence renal MCC development through other pathways independent of cAMP signaling. On the other hand, the reduction in renal MCCs in our morphants could also be due to increased cAMP levels by reduced blockage of adenylyl cyclase thanks to the deficiency of Cnr1. To investigate this, we utilized an adenylyl cyclase inhibitor previously used in zebrafish, SQ-22536, in tandem with the *cnr1* MO [55]. We generated *cnr1* morphants by injecting the *cnr1* MO at the single-cell stage and performed drug treatment with 0.01 mM SQ-22536 at the shield stage until 28 ss. Using the MCC marker *odf3b*, we observed a reduction in renal MCC numbers in *cnr1* morphants and *cnr1* morphants treated with 0.01 mM SQ-22536 (Figure 5D,E). Interestingly, WT embryos treated with 0.01 mM SQ-22536 also exhibited a slight reduction in MCC numbers, in line with our observation that a reduced cAMP level leads to reduced MCC development (Figure 5D,E). *cnr1* morphants, *cnr1* morphants treated with 0.01 mM SQ-22536 and WT treated with 0.01 mM SQ-22536 all exhibited an increase in embryos with a decreased MCC phenotype compared to WTs (Figure 5F). There was no significant difference in the number of *odf3b^+^* cells per nephron between *cnr1* morphants and *cnr1* morphants treated with 0.01 mM SQ-22536, suggesting that the effects of the *cnr1* MO and SQ-22536 on renal MCCs are likely redundant (Figure 5E). Taken together, our results suggest that *cnr1* likely also governs renal MCC development through distinct pathways apart from cAMP signaling.

## 4. Discussion

**Figure 6:**
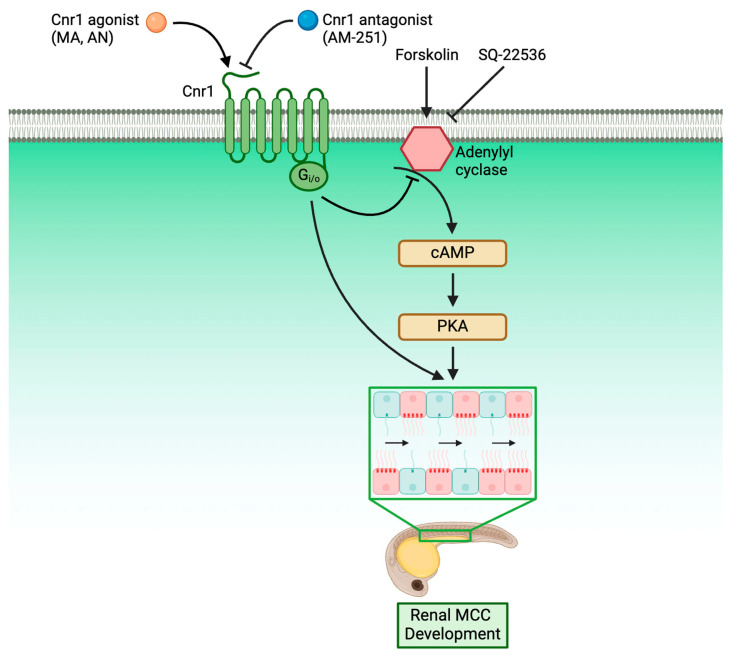
Roadmap delineating the relationship between Cnr1 and players in renal MCC development.

An understanding of the molecular genetics underlying cilia development is critical for our understanding of ciliopathies, as cilia are present across many tissues and are responsible for many critical diseases. Within the kidney, knowledge of renal cilia development is even more critical, as renal cilia defects are linked to many renal ciliopathies. However, the majority of research performed in renal ciliopathies has focused on genes and pathways regarding primary cilia [56,57,58]. Meanwhile, renal MCCs, while occurring in many renal disease conditions, have only been historically reported in patient biopsies without much understanding of the molecular genetics underlying their development [24,25,26,27]. In recent years, however, more and more studies have characterized MCC origin and revealed the complex genes and pathways underlying renal MCC development [28,41,42,43,59]. Our work here has further enriched the knowledge of the molecular pathways governing renal MCC development by identifying Cnr1 as a critical regulator for renal MCC development through the use of genetically deficient models and pharmacological approaches. Furthermore, we discovered that Cnr1 is essential for other ciliated tissues, including nasal placode, ear, KV and L/R patterning and neuromasts. We also showed, for the first time, that Cnr1 regulates renal MCC development through the cAMP signaling pathway, as well as independently (Figure 6).

The links between CB1 and kidney diseases, as well as its therapeutic potential for kidney diseases, have been recorded in the literature [1,20,60,61,62]. The majority of studies on the link between CB1 and kidney diseases have been on the over-expression of CB1 in renal conditions and how treatment with CB1 antagonists can reverse these defects. For example, CB1 expression was found to be highly upregulated in the glomeruli in diabetic nephropathy, as well as in renal fibrosis, acute interstitial nephritis and IgA nephropathy [17,62]. Similarly, the CB1 receptor was upregulated in patients with diabetic nephropathy, obesity-related glomerulopathy and focal segmental glomerulosclerosis [63]. In both diabetic mice and rats, the CB1 receptor was overexpressed in the kidney, including the proximal cells, glomeruli and podocytes [64,65,66]. Interestingly, the hybrid CB1 and iNOS antagonist MRI-1867 helped reverse obesity-induced CKD by improving the metabolic profile, kidney morphology and function, inflammation and fibrosis [63]. CB1 blockage by AM-251 ameliorated albuminuria- and diabetes-induced downregulation of podocyte markers [65]. AM-251 also attenuated apoptosis in a model of diabetic nephropathy induced by hyperlipidemia [67]. Similarly, CB1 antagonist SR141716 (also known as Rimonabant) improved insulin resistance and decreased albumin excretion and proinflammatory cytokines in diabetic kidneys [64]. SR141716 also alleviated increased proteinuria, creatinine clearance, renal hypertrophy and injury [68,69]. Pharmacological inhibition with AM-281 and SR141716 and genetic deletion of CB1 also alleviated renal dysfunction, oxidative stress, cell death and inflammatory response in the cisplatin-induced model of nephropathy [70].

In the present report, we provide new research on the use of CB1/Cnr1 antagonists and genetic deficiency in zebrafish kidneys. While our work also provided evidence that upregulation of *cnr1* using Cnr1 agonists lead to phenotypes associated with renal defects, such as pericardial edema, delayed PCT coiling and reduced renal MCC populations, we also surprisingly found that Cnr1 blockage using the antagonist AM-251 and genetic deficiency of *cnr1* also led to similar renal defects. We theorize that the reduction in renal MCCs observed in both agonist and morpholino/antagonist treatments could be due to divergent reasons. We have shown that reduced renal MCCs induced by Cnr1 agonists were successfully rescued by treatment with FSK, indicating cAMP signaling to be responsible for the phenotypes seen in agonist-treated embryos (Figure 5A–C). On the other hand, we found that treatment with SQ-22536 alone or with a cnr1 MO led to reduction in renal MCC populations (Figure 5D–F). These results indicated that the phenotypes seen in morphant- or antagonist-treated embryos are likely due to independent pathways. Furthermore, the genetic deficiency of *cnr1* also led to reduced cilia development across tissues. Our work thus calls for more diverse research on the use of CB1/Cnr1 antagonists and genetic deficiency as treatment, as we have revealed potential kidney defects in the zebrafish model through the use of both approaches.

Previous research has also shown cAMP signaling to be important for cilia development. In primary cilia, many components of cAMP signaling are present [71,72,73,74,75]. Additionally, many genetic factors are localized to the primary cilia, the loss of which causes disturbances in cAMP signaling, leading to ciliopathies and various metabolic defects [76,77,78]. In the kidney, regulation of cAMP is important in autosomal dominant polycystic kidney disease (ADPKD) and water balance disorders. Many genes that encode components of cAMP signaling pathways have been found to be expressed in many kidney tubule segments [79,80].

Previously, it has been shown that the loss of *ep4*, a GPCR that increases the level of cAMP, led to ciliogenesis defects like hydrocephalus, laterality defects and KV cilia reduction. However, the addition of FSK helped alleviate ciliopathies and restored KV cilia length [54]. On the other hand, overactivation of cAMP with FSK also caused renal cyst development [81]. It was also shown that the addition of cAMP and PKA activator led to an increase in primary cilia length [82]. While the majority of studies on cAMP signaling and cilia have been on primary cilia, our study revealed that cAMP signaling is essential for renal MCC development. Furthermore, we revealed that the reduction in the renal MCC population due to *cnr1* agonism could be rescued by FSK treatment, thus elucidating that Cnr1 regulates renal MCC development via cAMP signaling. Our research thus suggests the direction of using the cAMP signaling pathway to treat renal ciliopathies in kidney diseases, many of which exhibit excessive CB1 expression [17,62].

Future studies are necessary to further elucidate the role of CB1/Cnr1 in renal MCC development, particularly the relationship between Cnr1 and prostaglandin signaling in renal MCC development. The prostaglandin molecule PGE_2_ has been known to be upstream of the GPCR EP4 and cAMP signaling in regulating ciliogenesis [54]. Interestingly, a recent mouse knockout study with CNR1^−/−^ mouse showed an upregulation of PGC-1α, Cox1 and several markers of the prostaglandin signaling pathway [83]. Meanwhile, our lab has uncovered the relationship between many genetic factors and prostaglandin signaling in renal MCC development, thus indicating potentials for further studying the relationship between Cnr1 and prostaglandin signaling in renal MCC development [41,42,43,59]. Additionally, there is evidence on the activity of CB2 in several kidney conditions [84,85,86,87,88,89,90,91,92,93]. Previous studies also indicated that CB2 is expressed in hair cells and regulates cannabinol in ear hair cell development [94,95]. Therefore, it is possible that there might be a relationship between CB1 and CB2 and renal MCC development. Furthermore, there are many growing genetic factors that have been known to play an essential role in renal MCC development, with which CB1 could possibly interact [21].

Overall, our work has further elucidated the role of Cnr1 as a critical factor in renal MCC development and cilia development across organs. We showed that Cnr1 regulates renal MCC development through cAMP signaling, as well as independently. Overall, these findings have greater implications for the understanding of tissue ciliogenesis, renal ciliopathies and overall renal diseases at large.

## 5. Conclusions

Renal ciliopathy research is a vital area of ongoing study, and the important work of many investigators has delineated genes that play important fundamental roles in cilia development. However, the breadth and depth of studies on genes controlling renal primary cilia [58] has been much larger and more intricate than genes controlling renal MCCs [21,87]. Furthermore, while many signaling pathways have been implicated in kidney diseases, including endocannabinoid signaling and the Cnr1 receptor, there has been comparatively less knowledge concerning their relationship with renal cilia development. Through our study, we elucidated new insights into the roles of the Cnr1 receptor in renal cilia development, highlighting, for the first time, that Cnr1 regulates renal MCC development via cAMP signaling, as well as independently. We also highlighted the previously unknown role of Cnr1 in cilia development across zebrafish tissues. Our work thus further elevated our understanding of the role of Cnr1 in renal cilia, especially MCC development, further elucidating the connection between Cnr1 and the endocannabinoid signaling pathway as a whole to kidney diseases. Furthermore, our discovery that Cnr1 regulates renal MCC via cAMP signaling reveals the potential for future studies to explore and discover therapeutic treatments for renal ciliopathies.

## Data Availability

All data are provided in the paper or in the Supplementary Files.

## Supplementary Materials

The following supporting information can be downloaded at: https://www.mdpi.com/article/10.3390/jdb13020020/s1, Figure S1: Characterization of PCT coiling between treatments and the effect of *cnr1* deficiency on renal segments; Figure S2: Effect of *cnr1* agonism on renal segments; Figure S3: Effect of *cnr1* antagonism on renal segments; Figure S4: Loss of *cnr1* leads to reduced cilia in distal pronephros; Table S1: Reagents and resources used in this study.
