# Supplementary material for: Cannabinoid Receptor 1 Regulates Zebrafish Renal Multiciliated Cell Development via cAMP Signaling
Source: J Dev Biol. 2025 Jun 17;13(2):20. doi: 10.3390/jdb13020020 (PMC12194200; doi:10.3390/jdb13020020)
Supplement: Supplementary file 1 [file jdb-13-00020-s001.zip › jdb-3543369-supplementary.pdf]

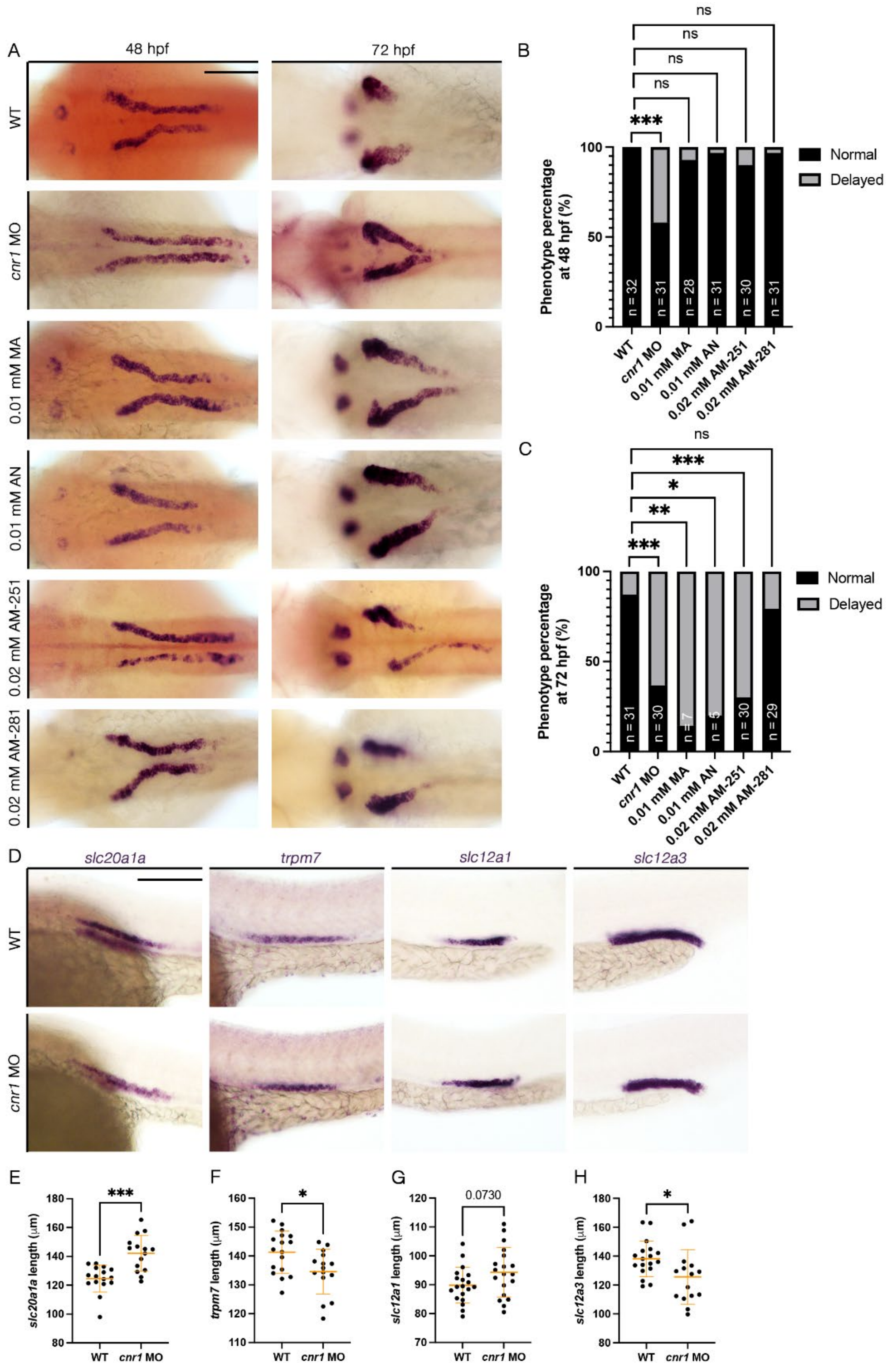

Figure S1. Characterization of PCT coiling between treatments and the effect of *cnr1* deficiency on renal segments. (A) 48 hpf and 72 hpf embryos between experimental groups stained via WISH using the mature PCT marker *slc20a1a*. Scale bar = 50 μm. (B,C) Phenotype

percentage of PCT coiling phenotypes at 48 hpf and 72 hpf. (D) 28 ss WT, *cnr1* MO stained via WISH using the segmental markers for PCT (*slc20a1a*), PST (*trpm7*), DE (*slc12a1*) and DL (*slc12a3*). Scale bar = 50 μm. (E-H) Domain length of *slc20a1a*, *trpm7*, *slc12a1* and *slc12a3* at 28 ss. Data presented on graphs are represented as mean ± SD; * $p<0.05$, ** $p< 0.01$ ***$p < 0.001$ and ****$p < 0.0001$ (Fisher's exact test with Holm's correction (Figure S1B,C) and t-test (Figure S1E-H)).

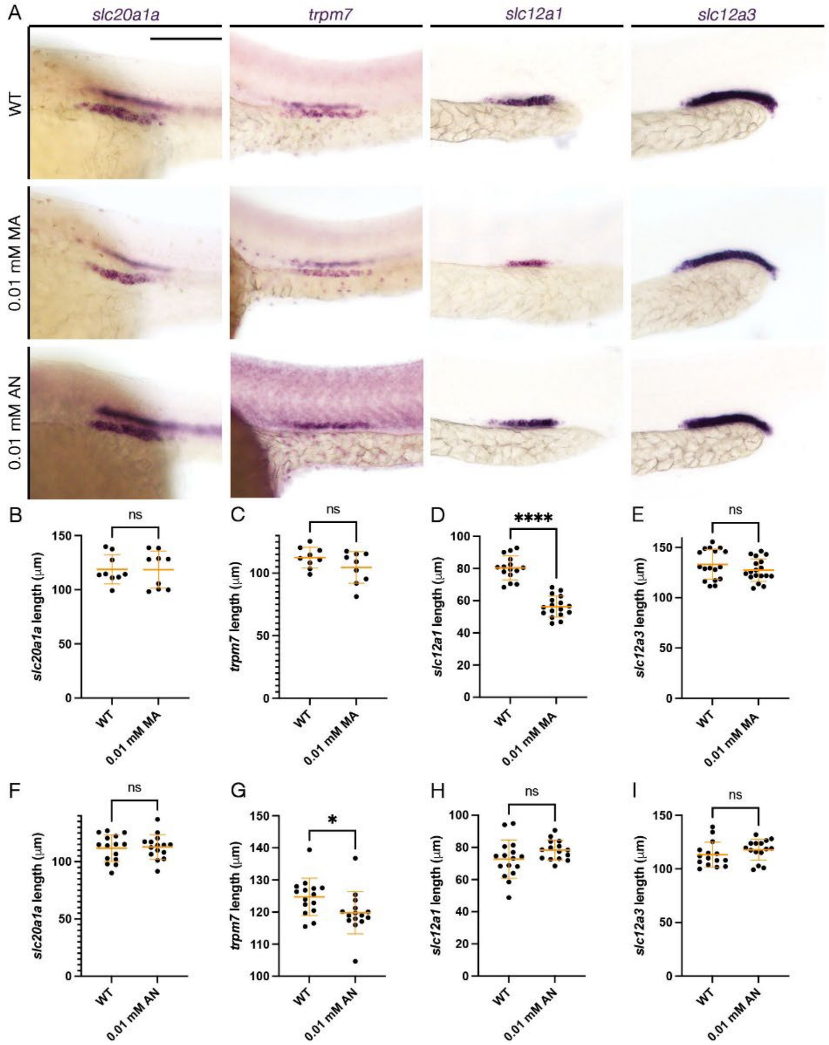

Figure S2. Effect of *cnr1* agonism on renal segments. (A) 28 ss WT, 0.01 mM MA and 0.01 mM AN-treated embryos stained via WISH using the segmental markers for PCT (*slc20a1a*), PST (*trpm7*), DE (*slc12a1*) and DL (*slc12a3*). Scale bar = 50 μm. (B-E) Domain length of *slc20a1a*, *trpm7*, *slc12a1* and *slc12a3* at 28 ss between WT and 0.01 mM MA-treated embryos. (F-I) Domain length of *slc20a1a*, *trpm7*, *slc12a1* and *slc12a3* at 28 ss between WT and 0.01 mM AN-treated embryos.

Data presented on graphs are represented as mean ± SD; * p<0.05, ** p< 0.01 ***p < 0.001 and ****p < 0.0001 (t-test).

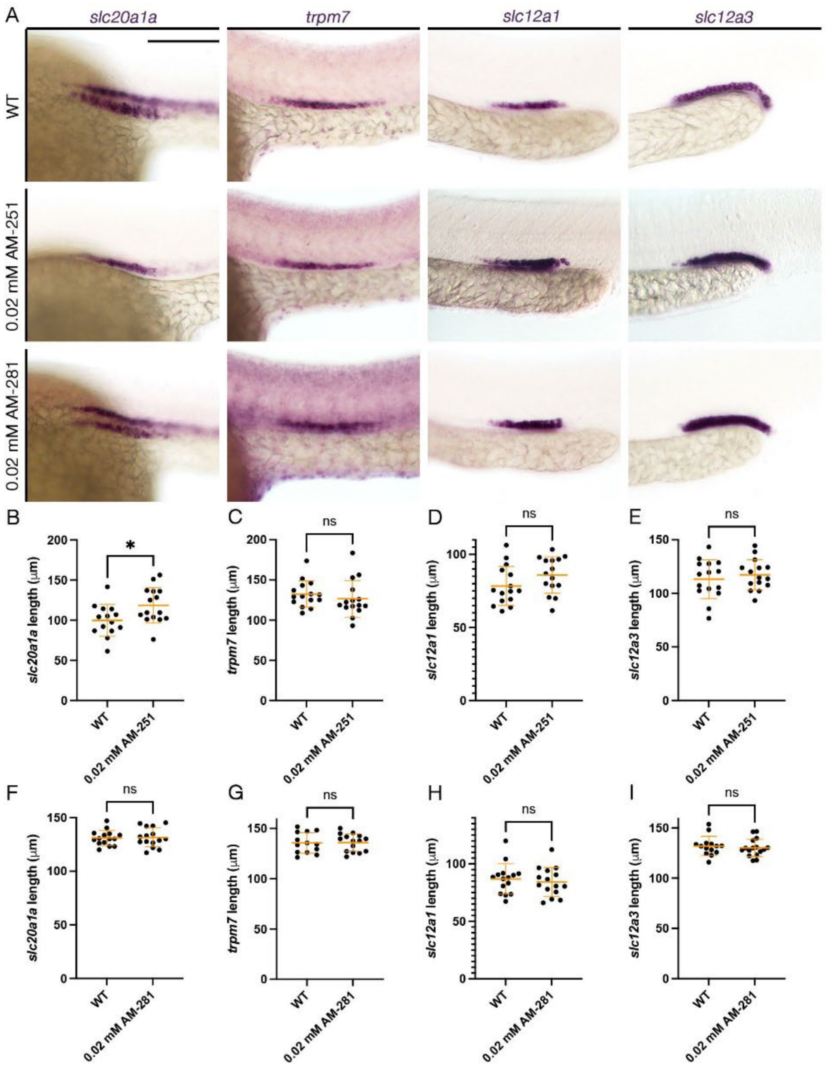

Figure S3. Effect of *cnr1* antagonism on renal segments. (A) 28 ss WT, 0.02 mM AM-251 and 0.02 mM AM-281 embryos stained via WISH using the segmental markers for PCT (*slc20a1a*), PST (*trpm7*), DE (*slc12a1*) and DL (*slc12a3*). Scale bar = 50 μm. (B-E) Domain length of *slc20a1a*, *trpm7*, *slc12a1* and *slc12a3* at 28 ss between WT and 0.02 mM AM-251-treated embryos. (F-I) Domain length of *slc20a1a*, *trpm7*, *slc12a1* and *slc12a3* at 28 ss between WT and 0.02 mM AM-281-treated embryos. Data presented on graphs are represented as mean ± SD; * p<0.05, ** p< 0.01 ***p < 0.001 and ****p < 0.0001 (t-test).

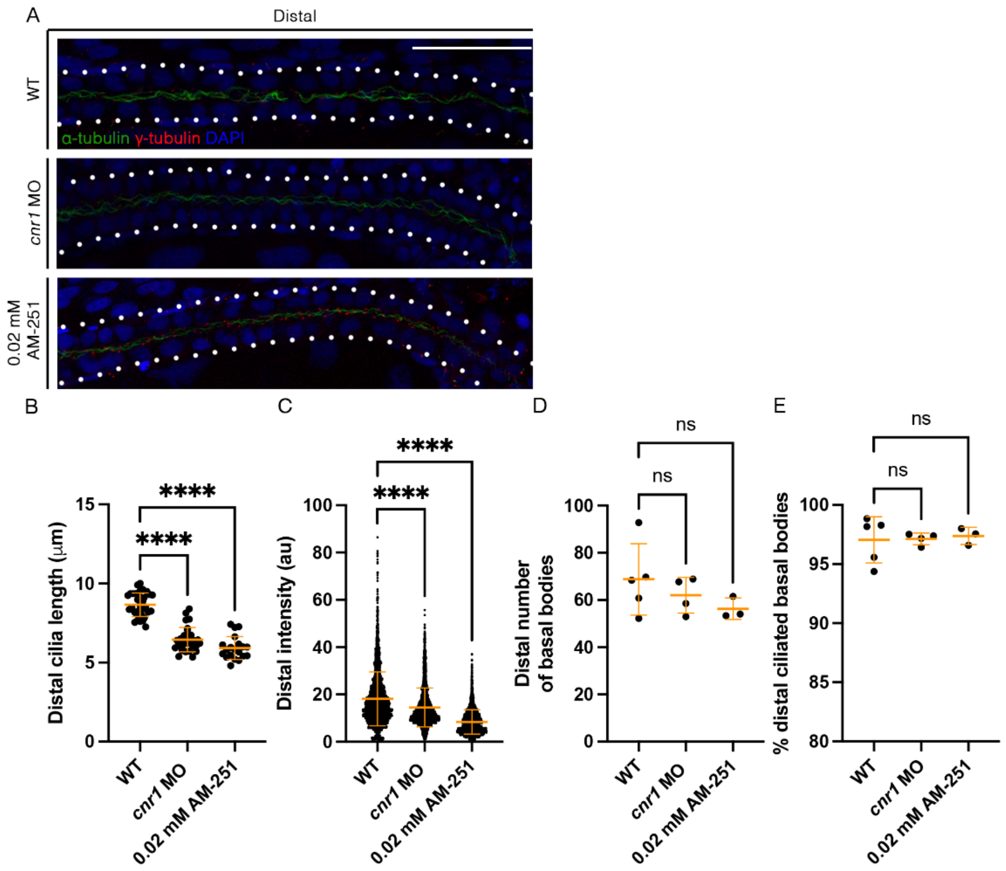

Figure S4. Loss of *cnr1* leads to reduced cilia in distal pronephros. (A) 28 hpf whole mount IF for acetylated α-tubulin (cilia, green), γ-tubulin (basal bodies, red), and DAPI (nucleus, blue) in the distal pronephros of WT, *cnr1* MO, and embryos treated with AM-251. Scale bar = 50 μm. (B) Distal cilia length at 28 hpf. (C) Fluorescence intensity plot of α-tubulin intensity in the distal pronephros at 28 hpf. (D) Number of basal bodies in the distal pronephros at 28 hpf. (E) Percentage of ciliated basal bodies/total basal bodies in the distal pronephros at 28 hpf. Data presented on graphs are represented as mean ± SD; * p<0.05, ** p< 0.01 ***p < 0.001 and ****p < 0.0001 (ANOVA).

Supplemental Table 1. Reagents and resources used in this study.

**Reagent or Resource**	**Source**	**Identifier**
**Antibodies**		
Anti-tubulin acetylated (cilia) - 1:400	Sigma-Aldrich	T6793; RRID:AB_477585
Anti-γ-tubulin (basal bodies) - 1:400	Sigma-Aldrich	T5192; RRID:AB_261690
Anti-PKC (apical surface) - 1:250	Santa Cruz	SC216; RRID:AB_2300359
Goat anti-Rabbit IgG (H + L) Highly Cross-Adsorbed Secondary Antibody, Alexa Fluor 594	Invitrogen	A11037; RRID:AB_2534095
Goat anti-Mouse IgG (H + L) Highly Cross-Adsorbed Secondary Antibody, Alexa Fluor 488	Invitrogen	A11029; RRID:AB_2534088
**Chemicals**		
Methanandamide	Enzo	BML-FA021-0025
Anandamide	Enzo	BML-FA017-0005
AM-251	Sigma	A6226
AM-281	Sigma	A0980
Forskolin	Sigma	F3917
SQ-22536	Sigma	568500
**Software**		

Fiji	http://fiji.sc/	RRID:SCR_002285
Prism v 10	GraphPad	https://www.graphpad.com/
